# A Recurrent Case of Mesothelial Pericardial Cyst

**DOI:** 10.7759/cureus.53522

**Published:** 2024-02-03

**Authors:** Jaafar A Hamdan, Akbar Khan, Annalee Mora, Amrita Randhawa, Maria Castano

**Affiliations:** 1 Internal Medicine, HCA Healthcare/University of South Florida (USF) Morsani College of Medicine: HCA Florida Oak Hill Hospital, Brooksville, USA; 2 Medicine, HCA Healthcare/University of South Florida (USF) Morsani College of Medicine: HCA Florida Oak Hill Hospital, Brooksville, USA

**Keywords:** benign cardiac mass, cardiac mass, chest pain, benign mesothelial cyst, pericardial cyst

## Abstract

This is a case of a 37-year-old female patient with past medical history of mitral valve prolapse and benign mesothelial pericardial cyst status post laparoscopic resection who presented to the emergency department with a chief complaint of right-sided sharp non-radiating chest pain due to recurrent case of benign mesothelial pericardial cyst. Though this is not a common pathology, it does overlap with common cardiovascular symptoms/conditions; not limited to but including chest pain, dyspnea, palpitations, pericardial effusions, infections and arrhythmias. It is crucial to have appropriate history and physical exam and appropriate evaluation to rule out pericardial cysts as well as their locations and their potential lethal mechanical implication on crucial nearby structures. This is significant in order to avoid uncommon but lethal cardiac complications in this condition such as cardiac arrhythmias, cardiac tamponade, right ventricular outflow tract and even sudden cardiac death.

## Introduction

Pericardial cysts are rare, benign congenital anomalies that originate from the mesothelial lining of the pericardium [1]. Though typically asymptomatic, these cysts can occasionally present with clinical manifestations, necessitating further evaluation and management. Clinically, pericardial cysts can present with chest pain, dyspnea, palpitations or fatigue [2]. These symptoms are non-specific and can be attributed to various cardiac or respiratory conditions. Pericardial cysts are generally benign and tend to have favorable prognosis [3]. However, in certain cases, complications can arise due to their size, location, or mechanical impact on surrounding structures. Complications include compression of adjacent structures, pericardial effusions, infection, or arrhythmias [2,4]. Initially, these cysts may go undetected on imaging, including computed tomography (CT) [1,5]. There is also the possibility of recurrence; therefore, a thorough evaluation is necessary to establish an accurate diagnosis and differentiate pericardial cysts from other etiologies [1]. 

## Case presentation

A 37-year-old female patient with a past medical history of reported mitral valve prolapse but without positive echocardiogram (ECHO) findings and pericardial cyst status post laparoscopic resection eight years ago presented to the emergency department with a chief complaint of chest pain described as sharp, non-radiating, on a severity of 8/10, no worsening or alleviating factors. The patient was admitted on a different occasion with similar symptoms. On the current visit, the patient presented with worsening symptoms. On initial encounter; blood pressure 127/76 mmHg, pulse 88, respiratory rate 16, oxygen saturation 99% on room air, temperature 36.5C. Physical exam findings; S1 and S2 appreciated, regular rate and rhythm. A systolic murmur was appreciated on auscultation and no rubs/gallops. Chest wall tenderness with palpation was present. Below in Table 1 are abnormal lab findings on the initial encounter.

**Table 1:** Abnormal laboratory values on admission at the initial encounter.

Parameter	Values	Reference values
White blood count (10>3/uL)	11.8 uL	4.0-10.5 (10>3 uL)
Eosinophil %	0.5%	0.7-5.8 %
Neutrophil #	7.68	1.56-6.13 (10>3 uL)
Monocytes #	0.87	0.24-0.63 (10>3 uL)
Activated Partial Thromboplastin Clotting Time	24 seconds	25-38 seconds
Anion Gap	5 mmol/L	7-16 mmol/L

**Figure 1:**
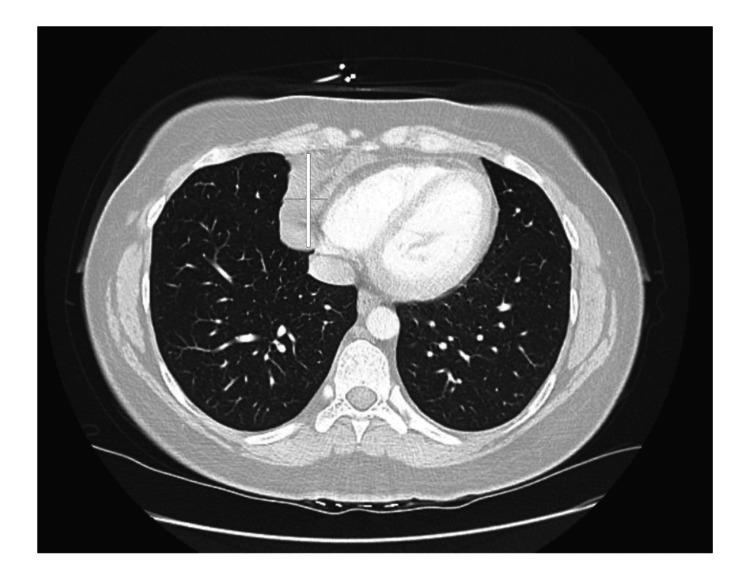
Computed Tomography Angiography (CTA) chest axial view. An axial CTA view of a pericardial cyst fluid in the lower anterior mediastinum indicated via the vertical and horizontal lines. Measurement about 6.0 x 5.4 x 6.4cm.

EKG showed pulse 83, normal sinus rhythm, normal heart axis, and no signs of ischemia/infarction. On chest X-ray (CXR), there were no acute cardiopulmonary pathologies. On CT chest angiography with contrast (CTA), there was a 6.0 x 5.4 x 6.4 cm fluid density structure present in the lower anterior mediastinum (Figure 1). 

Located adjacent to the pericardium without inflammatory characteristics, suggestive of recurrent cystic lesion. Records were obtained from a previous medical facility where the patient had a previous pericardial cyst resection done eight years ago, an 8.0 x 3.5 x 0.6 cm cystic structure which was a benign mesothelial cyst of pericardium per surgical pathology report. Initially, the mass was excised laparoscopically and eight years later, the recurrence was at the same anatomical place.

During hospital stay, ECHO showed an ejection fraction of 60% and right ventricular systolic pressure (RVSP) of 32.2 mmHg. Both cardiothoracic and interventional cardiology were consulted. Initially, a needle biopsy was to be done via cardiothoracic surgery to further analyze the nature of the mediastinal mass. However, on further review it was decided that the patient obtain pulmonary function tests (PFTs) outpatient and follow up for re-evaluation for surgical intervention if warranted as she was experiencing intermittent shortness of breath. Secondly, on further discussion, it was decided patient would benefit from a coronary computed tomography angiography (CCTA) to assess the anatomy of coronary arteries and the pericardial cyst. CCTA showed a benign-appearing right pericardial cyst, no enhancing mass, pericardial thickening, or pericardial effusion. Otherwise normal CT of the heart and coronary arteries. The patient was then stable for discharge and was to follow up outpatient with cardiothoracic surgery for further evaluation/treatment. 

## Discussion

Pericardial cysts are rare, benign lesions in the intrathoracic region that account for 6%-7% of all mediastinal masses [6,7] with an incidence of 1 in every 100,000 people [8]. Most patients present asymptomatically as a solitary mass from the diaphragm to the superior mediastinum. Our patient's cyst is located in the lower anterior mediastinum, which can be seen in 8% of cases [8]. Histologically, the cysts contain serous fluid with a single layer of mesothelial cells [7]. Most cases are detected incidentally on chest radiographs for other reasons; however, these can cause significant growth that affects surrounding tissues, leading to clinical symptoms parallel to our patient's presentation of chest pain and dyspnea. Although she had the resection done eight years ago, there is a potential recurrence rate of 33% over time [9]. 

Incidental findings on the initial CXR warranted further imaging studies such as CT. Our patient's chest CT scan showed a fluid density structure consistent with a cystic lesion. CT scan typically shows a homogenous, single, thin-walled, and sharply defined mass without septation or solid component [8]. It provides anatomic details and characterizations to aid further decisions for surgical intervention if warranted. It also differentiates other etiologies of neoplasms and aneurysms [10,11]. Similarly, echocardiography is a non-invasive test that offers the cyst's exact location and assesses the heart's functionality. Our patient had a 60% ejection fraction without evidence of a pericardial cyst compressing the pericardial sac. CCTA performed was unremarkable, however was able to identify the presence of a pericardial cyst. 

Management of pericardial cysts follows a systematic approach. Asymptomatic patients can be monitored periodically with a CT scan and conservative management. However, symptomatic patients can undergo minimally invasive percutaneous aspiration or surgical resection [8] depending on the location, risk factors of the patient, and characteristics of the cyst [11] to prevent life-threatening conditions as a result of compression of adjacent vital organs. Our patient was evaluated by the cardiothoracic surgery team for the possibility of surgical intervention. However, no emergent interventions were required as the tests performed did not reveal the cyst compressing any surrounding structures. A pulmonary function test was advised to be done outpatient to rule out any lung diseases as she had dyspnea at times. Periodic surveillance was highly recommended as persistent symptoms can possibly result from the cyst's direct communication into the pericardium and pericardial sac, causing chest pain and dyspnea. Similarly, an impingement over the atria and pulmonary vessels can cause tachycardia, palpitations, or arrhythmias. 

## Conclusions

Pericardial cysts are a rare, benign congenital abnormality, originating from mesothelial lining of the pericardium. Though it is asymptomatic the majority of the time, it can occasionally present with clinical manifestations, necessitating further evaluation and interventions. A significant aspect to keep in the equation is pericardial cysts can present with chest pain, dyspnea, or palpitations; hence, it is crucial to have patient symptoms properly evaluated and managed even if there is a known history of pericardial cyst. In addition, even with an established pericardial cyst diagnosis, it is important to intervene when symptoms are present as complications including compression of adjacent structures, pericardial effusions, and cardiac complications such as arrhythmias can ensue. 
